# Feasibility of community-based control of tsetse: A pilot project using Tiny Targets in the Democratic Republic of Congo

**DOI:** 10.1371/journal.pntd.0008696

**Published:** 2020-09-24

**Authors:** Catiane Vander Kelen, Alain Mpanya, Marleen Boelaert, Erick Miaka, Dennis Pérez Chacón, Justin Pulford, Richard Selby, Steve J. Torr

**Affiliations:** 1 Institute of Tropical Medicine, Antwerp, Belgium; 2 Programme National de Lutte Contre la Trypanosomiase Humaine Africaine, Kinshasa, Democratic Republic of Congo; 3 Instituto de Medicina Tropical Pedro Kourí, Havana, Cuba; 4 Liverpool School of Tropical Medicine, Liverpool, United Kingdom; Universidad de Buenos Aires, ARGENTINA

## Abstract

Gambiense Human African Trypanosomiasis (g-HAT) is a neglected tropical disease caused by trypanosomes transmitted by tsetse flies. 70% of cases in 2019 (604/863) occurred in the Democratic Republic of Congo (DRC). The national programme for g-HAT elimination in DRC includes a large-scale deployment of Tiny Targets which attract and kill tsetse. This intervention is directed by vector-control specialists with small teams, moving in canoes, deploying Tiny Targets along riverbanks where tsetse concentrate. While the targets are deployed in communal areas, and the method is cheap and easy-to-use, local people have little involvement. This study aimed to evaluate if a community-led vector control programme was feasible in the context of DRC’s g-HAT elimination programme. In 2017, a community-led intervention was implemented in three villages in the Kwilu province of DRC. This intervention was evaluated through an Action Research with qualitative data collected through 21 focus group discussions and 289 hours of observation. Also the geographical location and quality of each Tiny Targets were collected (total number deployed = 2429). This research revealed that community-based approach largely worked: people were motivated and proactive, showed a good application of the acquired knowledge resulting in an effective deployment of Tiny Targets. In addition, our study provided evidence that acceptability of the targets by the community can improve deployment quality by reducing target loss and damage. The approach was feasible in places where canoe-based teams could not reach. Against these advantages, a community-based approach was time-consuming and had to adapt to the seasonal and daily rhythms of the community. A community-based approach for tsetse control is technically feasible and recommended but limits to the speed and scale of the approach restraints its application as a standalone strategy in a large-scale national programme aiming to eliminate g-HAT in a short timeframe.

## Author summary

Sleeping sickness (Human African Trypanosomiasis, HAT) is a neglected disease caused by *Trypanosoma brucei gambiense* or *T*.*b*.*rhodesiense* both transmitted by tsetse flies. Currently 70% of the global cases of Gambiense HAT (g-HAT) occur in Democratic Republic of Congo (DRC). Over the last 20 years, a national programme of screening and treating humans has driven down the number of cases of g-HAT from >30,000 to <2,000 cases/year. However, epidemiological models suggest that the complete elimination of g-HAT across DRC requires the addition of vector control. Accordingly, in 2016 large-scale deployment of Tiny Targets which attract and kill tsetse, was adopted as part of the national strategy to eliminate g-HAT. Targets are deployed by canoe-based teams directed and managed by vector control specialists. Using an Action-Research approach and qualitative methods, we assessed whether local communities in three villages could play a greater role. We found that Tiny Targets were well accepted by local people who deployed targets effectively, but the speed and scale of the deployment was limited by the underlying movement of local people and the rhythms of their life. We conclude that a community-based approach is feasible but not as a standalone large-scale programme in DRC.

## Introduction

Human African trypanosomiasis (HAT), also known as sleeping sickness, is a fatal disease caused by *Trypanosoma brucei gambiense* or *T*.*b*.*rhodesiense* both transmitted by tsetse flies. It is a public health problem unique to sub-Saharan Africa where it affects mainly poor and rural populations. Gambiense HAT (g-HAT) is an anthroponotic disease with a relatively slow progression. Rhodesiense HAT (r-HAT) is an acutely progressing zoonotic disease with both wild animals and livestock, acting as reservoir hosts [1].

**Fig 1:**
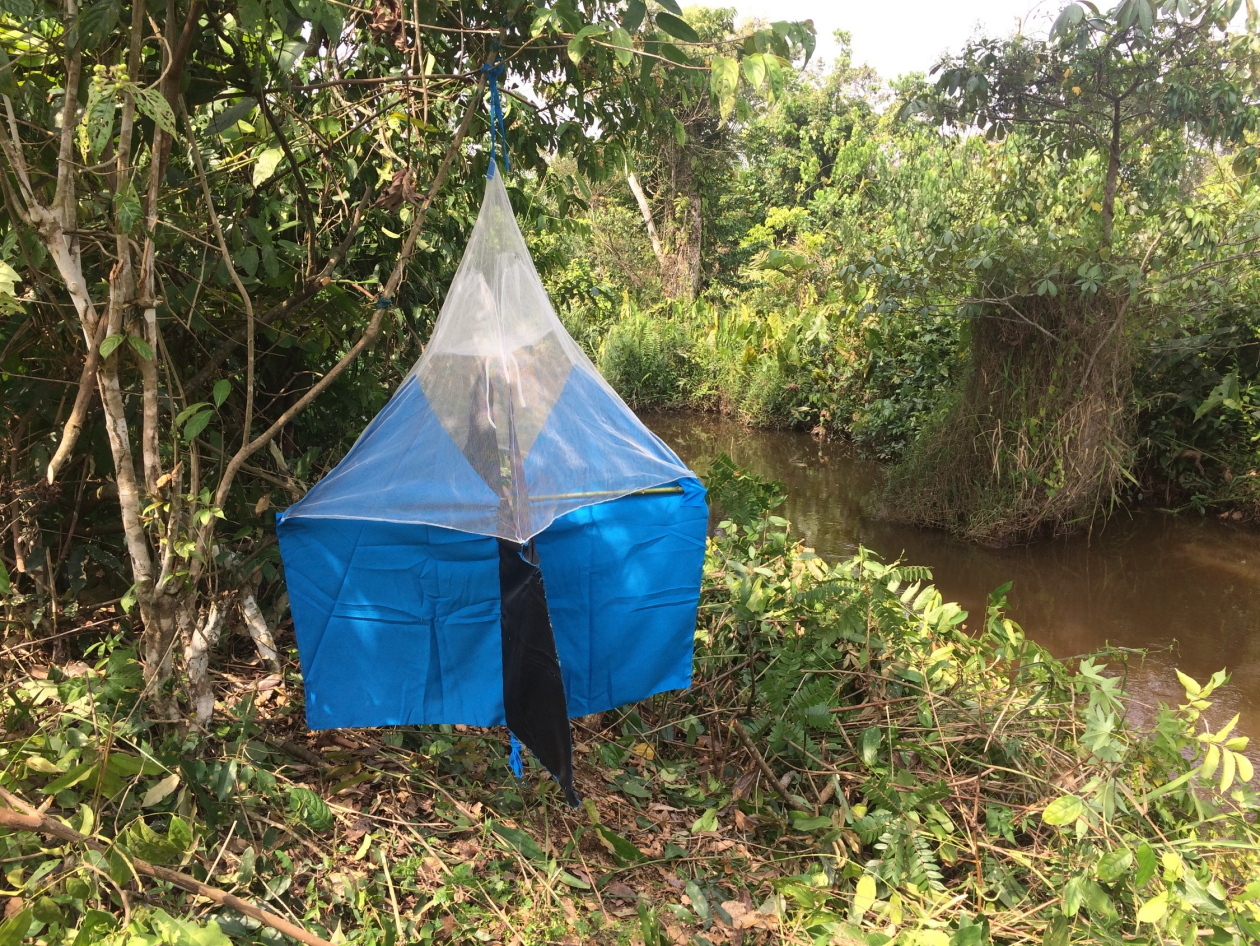
Tsetse Trap deployed to measure fly density before Tiny Targets deployment. (Credit: ST).

**Fig 2:**
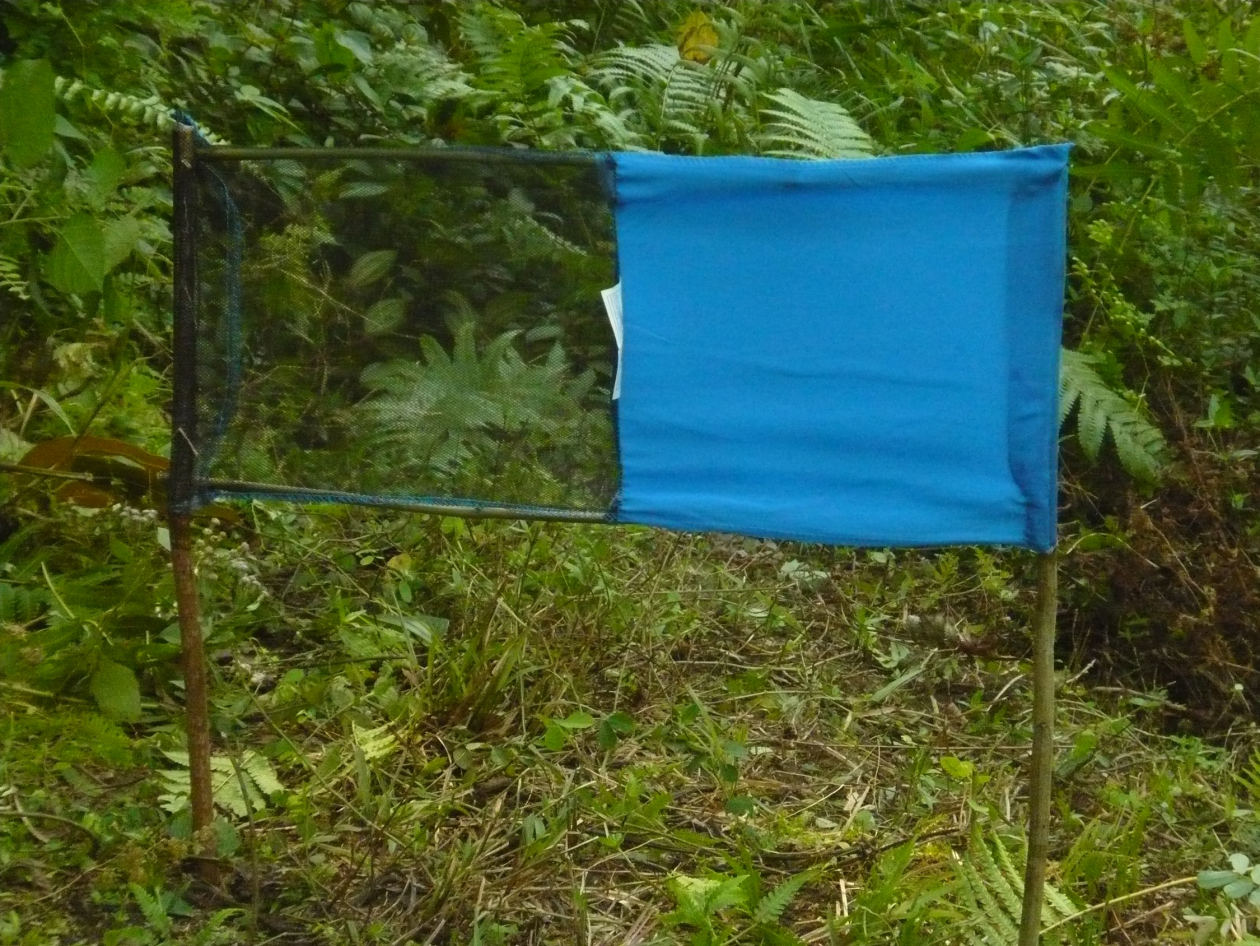
Tiny Target deployed by a Kisoko committee member. (Credit: CVK).

Gambiense HAT accounted for more than 88% of the reported cases in 2019 (863/979) [2]. The Democratic Republic of the Congo (DRC) is the country most affected by g-HAT, reporting 70% of all cases in 2019 (604/863) [2]. In 2012, the World Health Organization (WHO) presented a plan to eliminate HAT, first by reducing its incidence to very low levels by 2020 - (<1 new case/year/10,000 people in 90% foci and < 2000 cases reported globally)—and, in a second stage, interrupting transmission by 2030 [3, 4]. Active case detection and case treatment is the mainstay of efforts against g-HAT. The approach has led to a massive decrease in the DRC burden of g-HAT from 16,951 cases in 2000 to just 604 in 2019 [2]. Despite this achievement, models predict that sole reliance on active screening and treatment strategy will not achieve to interrupt transmission by 2030 [5]. In persistent endemic areas, HAT detection rates during active screening may be lower than 50% of the actual existing cases [1] resulting in continuing transmission within these communities [6–9]. Mathematical models suggest that in these foci, the 2030 elimination goals can be achieved by adding vector control to screening and treatment [5]. Recently developed Tiny Targets, small (50 x 25 cm) panels of cloth impregnated with insecticide, are more cost-effective at controlling tsetse than pre-existing methods, including use of traps (Figs 1 and 2) [10, 11]. Tiny Targets have already been used effectively in Uganda, Guinea, Ivory Coast and Chad [1, 12, 13].

Tiny Targets were first used experimentally in DRC in 2014. The Tiny Targets deployment strategy was based on method used in successful operations conducted in, Chad and Uganda [1, 13, 14] with targets being deployed at 50 m intervals every six months in tsetse riverine habitat [1]. The impact of targets on tsetse populations is assessed using a sparse network of monitoring traps. In DRC this strategy has been equally successful, despite the approach having to be slightly modified. Modification was due to road networks being poor throughout DRC’s remote endemic areas but the large navigable rivers present providing access by which canoe-borne teams could deploy targets in the tsetse’s habitat.

In the first phase, Tiny Targets deployment in DRC was “expert-led”, meaning that professional entomologists planned, organised and implemented the activities employing local workers. However, the simplicity of Tiny Targets makes them a strong candidate for a community-based (CB) approach.

Small and medium-scale studies involving community participation in trap-based tsetse control efforts were carried out in the 1980s and 1990s [15–18]. Several studies showed that the involvement of communities could improve acceptability and efficacy while reducing costs. However, community involvement was primarily seen by programme planners as a cost-cutting solution. Community participation and adherence was expected without consideration of community priorities and over time donor commitment to participatory projects for vector control in HAT has dwindled [18, 19]. CB-approaches normally prioritise community participation with an empowerment objective [20, 21]. In practice, this means activities are planned, organised, implemented and monitored by the communities themselves and adapted to community rhythm. More recent examples of CB-approaches to control the vectors of HAT are rare, seemingly limited to an active community participation project in South Sudan which used traps [22] and a pilot study of a community-led tsetse control intervention using Tiny Targets in Uganda. The study in Uganda, conducted by Kovacic *et al*. in 2015, concluded that not only was this approach valuable from a cost aspect but also that communities showed great capacity, acceptance and motivation to lead a tsetse control project. Kovacic recommended testing community-based approach in other contexts [13].

The aim of this study was to assess the operational feasibility of a community-led tsetse control strategy using Tiny Targets in DRC, evaluating whether this approach could form a part of the national strategy for HAT elimination.

## Material and methods

### Ethics statement

The research protocol was reviewed and approved by the ethical committee of the Institute of Tropical Medicine in Antwerp, Belgium (ref: 1157/17) and the public health school of the University of Kinshasa (ref: ESP/CE/029/2017). Authority of Yasa Bonga health district and village chiefs were informed and gave permission to conduct the study prior to data collection. All participants were informed about the objective of the research, their voluntary participation and their right to withdraw from the study. Oral consent was audio recorded.

### Study area

In 2016, an expert-led vector control project using Tiny Targets was initiated in two health districts (Yasa Bonga and Mosango) in Kwilu province, part of the former Bandundu province, the most affected HAT province in DRC. The recently designated province of Kwilu is located to the east of the capital Kinshasa. Apart from the capital Bandundu and the town of Kikwit, the province is rural. The Kwilu province is divided into 19 health districts which are further divided into health areas. The study sites were in the health district of Yasa Bonga. Yasa Bonga covers of 2,810 km^2^ with an estimated population of 180,439 across 20 health areas [23].

**Fig 3:**
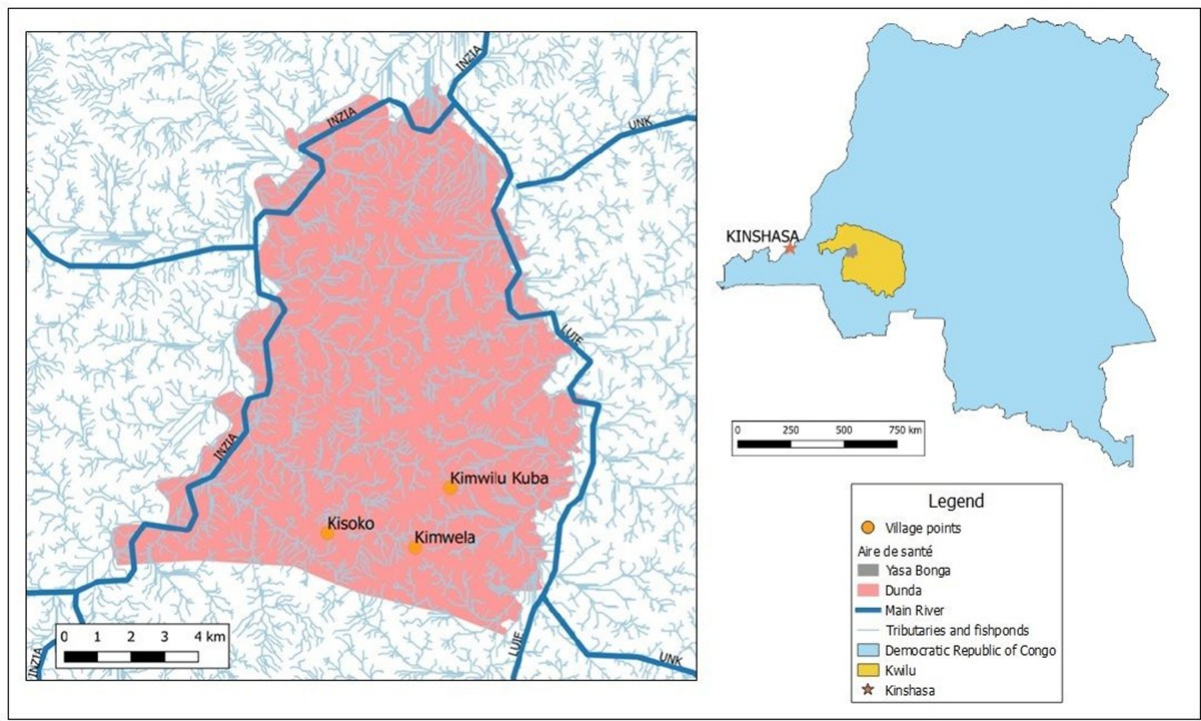
Location of the three pilot villages in the Dunda health area, Yassa Bonga health zone, Kwilu provinceplus de main rivers, tributaries and fishponds (Original work:CVK & RS).

The study was launched in 2017, to determine the feasibility of a CB- approach for the Tiny Targets deployment, which was fully organised and managed by local community members. Three pilot villages were selected from which 10 cases of g-HAT had been reported in the five years previous to our research: Kimwilu Kuba (Lat: -465.413; long:17.68.374; Pop: 1441; Cases: 4), Kimwela (Lat: -46.705; Long: 1.767.604; Pop: 1200; Cases: 3) and Kisoko (Lat: -439.925; Long: 1.739.231; Pop: 1005. Cases: 3) in the Dunda health area in the south west of the Yasa Bonga health district. Dunda has a total area of 135 km^2^ between the rivers Inzia and Luie (Fig 3). Dunda has a total population of 9,602 inhabitants from 11 villages and between 2013 and 2017, 25 cases of g-HAT were reported. Prior to this study, Tiny Targets had not been deployed anywhere in Dunda [24] [25].

### Study Population

**Fig 4:**
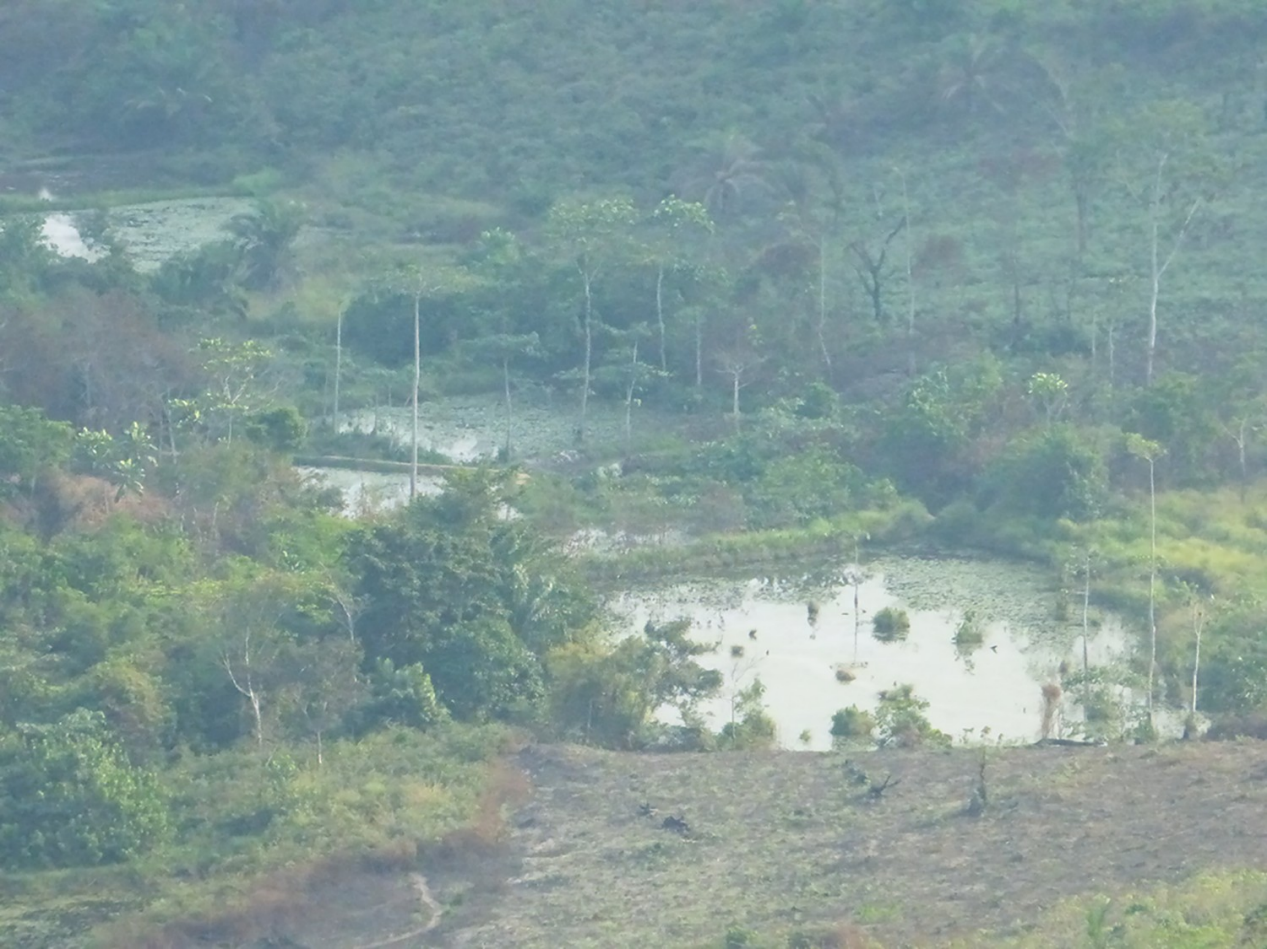
Human-Made Fishponds in Yasa Bonga Health Zone. (Credit: CVK).

Dunda’s population are largely sustained through agriculture and aquaculture in ponds made by sequentially damming tributaries that feed into the main rivers Luie and Inzia (Figs 3 and 4). This practice conserves natural riverine vegetation along the margins of the ponds and provides a favourable environment for tsetse flies. In Dunda, villages are generally situated on the top of small hills while activities related to aquaculture take place in the valley surrounding each village. Based on the geographic and population situation it was decided that the CB-approach would prioritise Tiny Targets deployment around the fishponds areas where tsetse flies bite the community members, so-called ‘contact points’, instead of the main riverbanks that are not often frequented and are hard to reach on foot.

In Kwilu, villages are made up of several different clans. In the three pilot villages, the clans’ number were six (Kimwilu Kuba) twenty (Kimwela) and seven (Kisoko). Each clan is represented by a traditional chief called a ‘*notable’*. “*Notables”* are hierarchically dominated by the village chief who represents the main authority of each village. The number of clans varies in function of territorial divisions, including ponds networks, which are constantly changing according to land conflicts and heritages claims.

### Implementation of pilot project

The pilot took place over 15 months between July 2017 and September 2018 allowing three rounds of target deployment. The first deployment and its preparation lasted from July 2017 to end of September 2017 and involved three key steps: 1) the creation of vector control committees, training, organisation and planning 2) assembling and deployment of Tiny Targets 3) data collection and evaluation.

We adopted an action-research methodology which is participatory by nature and aims to generate an action or a change as well as studying it [26]. The research team operating in the field included two Kikongo-speaking Congolese anthropologists (one male, one female) with previous experience in social mobilisation while supervised by the principal investigator (CVK). The research team initiated the creation of vector control committees in each selected village, offered training and support by providing information, dissemination of good practices along with technical and didactic material (T-shirts, awareness material, boots, machetes, Tiny Targets and traps) and encouraged communities to progressively assume leadership of the project taking on organisation and management of the vector control activity. Table 1 lists all field activities that benefited from face-to-face support by the research team in order to empower the committees. The face-to-face activities were divided across 12 mentorship sessions held with each village committee (36 in total) each lasting 2 to 3 hours, taking place at the villages’ school sports pitch. Mentoring sessions consisted of training and preparation or evaluation meetings. In addition to mentorship sessions, the assembling and deployment of Tiny Targets was supported by the research team through field visits: eight visits per community for the first deployment, six for the second and no visit for the third. Committee members participated voluntarily without financial compensation

**Table 1:** List of all field activities that benefited from face-to-face support by the research team.

Phases	Research team’s mentoring sessions	Timeframe
**Preparation**	1. **Meeting with chiefs**	July-August 2017
	2. **Meeting with committee members**	
	3. **HAT training**	
	4. **Preparing awareness raising for the rest of the community**	
	5. **Tiny Target training**	
	6. **Activity planning**	
**First Tiny Targets deployment**	*Field visits from the research team during assembling and Tiny Targets deployment (6 field visits per communities)*	August 2017
**After the first Tiny Targets deployment**	7. **Putting Monitoring System in place and indicators**	October 2017
	8. **Evaluation: how to improve?**	
**Other Tiny Targets deployments**	9. **Second Tiny Targets deployment preparation**	February 2018
	*Field visits from the research team during assembling and Tiny Targets deployment (6 field visits per communities*	February 2018
	10. **Evaluation: How to improve?**	February
	11. **Third Tiny Targets deployment preparation**	July 2018
	12. **Evaluation: How to improve?**	September 2018

The research team presented the tsetse control plan to the chief of each village in the first instance. All three village chiefs supported the proposed tsetse control plan, acknowledging the harmful presence of HAT in their respective villages. However, the three village chiefs refused the research team request to organise more formal meetings with all traditional chiefs and community leaders to ensure the relevant information reached every community subgroup (age, gender, clans…). Subsequently, to ensure CB-approach principles, the research team explained to the chiefs how important it was the participants were truly interested, that it was open to everybody, not only to the usual and “formal” community health workers. The research team requested that women should be encouraged to participate. All participants should be volunteers who would not receive any financial benefits. Each of the three village’ chiefs then selected a Vector Control Committee of 12–17 people composed of both genders.

The research team first trained Vector Control Committees on HAT and vector control during two sessions (one theoretical and one practical) with interactive and dynamic training methods (e.g., visuals, games, discussion, drawing, practical use of targets). Each Vector Control Committee then raised awareness in the community using didactic tools (images and examples of Tiny Target) either by going from door to door or holding small-group discussions at churches or schools. Afterwards, Vector Control Committees held planning workshops to determine the places to deploy, the number of targets that will be deployed and the division of work. Before each Tiny Targets deployment, they placed tsetse traps in strategic points to collect data on fly density. Committees then assembled and deployed the Tiny Targets, organised a monitoring system to monitor the deployment and participated in all the evaluations. All activities took place during committee members’ spare time so as not to interfere with field labour or other economic activities. Table 2 summarises the main activities the committees took responsibility for.

**Table 2:** Main responsibilities taken by the Vector Control Committees after mentorship.

**1**	Committees human resources management
**2**	Identification of tsetse hot spots
**3**	Maintenance of awareness about Tiny Targets in the village
**4**	Deployment of traps to measure tsetse density before each Tiny Targets deployment
**5**	Organisation, planning and work assignment for each Tiny Targets deployment
**6**	Maintenance of Tiny Targets
**7**	Data collections: Observations on efficiency
**8**	Evaluation: success, challenges and solutions.

### Data collection

To assess operational feasibility for CB tsetse control, data were collected on three principle aspects: 1) establishment and maintenance of a CB- organisational structure, 2) Tiny Target implementation, and 3) maintenance and evaluation.

Data were collected through 289 hours of observation during mentoring sessions and field visits recorded in a notebook, photographically and via audio-recorded mixed gender focus group discussions (FGDs) held with committee members before or after mentoring sessions. A total of 7 FGDs (21 in total) were conducted within each village across the study period. Each FGD had a specific topic including: Motivation factors, perception of tsetse nuisance, gender participation and perception of project impact and community empowerment. Quantitative data collection tools were also used (Global Positioning System and a Tiny Targets quality evaluation grid).

To assess establishment and maintenance of a community-based structure, information was collected through FGDs, observation and mentoring sessions. The data explores the committee group constitution, the group functionality and the motivating or demotivating factors of the committee members to be part of the project and the participation evolution.

Implementation was assessed through qualitative and quantitative methods. Observation and FGDs were used to study how the committee applied the acquired knowledge into practice. In addition, to determine if the implementation was being effective, an external team assessed each deployed Tiny Target in terms of its placement and quality. Placement data were collected using a Global Positioning System (GPS). Quality was assessed using a pre-determined observational grid based on four criteria: (i) deployed in habitat suitable for tsetse, (ii) 15–20 cm above the ground, (iii) vegetation had been cleared around the targets, (iv) the target was correctly assembled on its wooden frame. If any of the criteria was not fulfilled the targets was considered dysfunctional. We attempted to measure fly density reduction by placing traps during 48 hours before each Tiny Targets deployment. Not surprisingly, considering the tsetse catch rate in general [27], the catch of tsetse in the small territory of the study sites was too low (total catch = 6 tsetse) to permit statistical analysis as a result the impact of targets on tsetse population is not considered in this paper.

Finally, maintenance and evaluation systems were collected qualitatively through observation during mentoring meetings and FGDs held after deployments. Data were collected on post-implementation aspects: data collection capacities and Tiny Targets maintenance organisation.

### Data analysis

Audio-recorded FGDs were translated from Kikongo to French and transcribed by external translators. Field notes were systematically reported in a field note journal after each interaction with communities. All the transcripts and field notes were cross-checked by the research team to ensure accuracy. CVK analysed the data using a thematic content analysis approach [26]. This analysis method combines a deductive approach, through predefined themes in the FGDs questions, meetings or observational guides, and an inductive approach for which themes were identified through careful reading and re-reading of the data, and if patterns were recognized, these emerging themes became the categories for analysis. NVivo software (version 11; QSR International, Melbourne, Australia) was used to conduct the data analysis.

## Results

### Establishment and maintenance of a community-based organisational structure

#### Committees composition gender balance and lack of community representation

Chiefs organised the selection system to create the committees, but despite encouragement from the research team, women were under-represented. Of the 45 members across the three committees, only 12 were women. Table 3 shows the gender composition amongst committee members.

**Table 3:** Gender representation amongst committee members.

Villages	Committee’s names	Number of male participants	Number of female participants	Total participants
**Kimwilu Kuba (KK)**	“The willingness”	10	2	12
**Kimwela (KA)**	“The tsetse hunters”	12	5	17
**Kisoko (KO)**	“The tsetse enemies	11	5	16

This gender inequality could be explained by different factors. Women reported it was because this type of project was generally for men and many women did not think it was meant to include them. Also, it was reported that female participation in this kind of project increases their workload due to their other gender-based roles.

“We suffer quite a lot with field work and in addition with the project activities. Especially, us, the women. We have to do our field work like the men and then we also have to prepare a meal for the afternoon, take care of children… And on top of all that, we must take care of the Tiny Targets and we don’t get paid… This discouraged women to join.” (FGD, KK, October 2017)

The committees not only lacked community representation regarding gender but also in other aspects including clan, ethnic group and religion. For example, in Kimwilu Kuba all the participants were part of the same religious group as the chief (*l’église des noirs*), while this village has four different official religions. However, this lack of diversity did not necessarily result in inequities during the implementation; committee members paid particular attention to include putative transmission hot spots frequented by woman, elderly and all the clans.

#### Committee functionality: Innovation and adaptation

Throughout the study period, the three committees functioned without the guidance of the research team on most occasions. After the training phase, they showed clear skills in organisational initiative, decision making, innovative ideas and finding solutions to operational problems. For example, they took the initiative of organising awareness sessions using different techniques such as visiting people in their homes, at church or markets. Also, at the end of the first deployment, communities took the initiative of establishing a “Tiny Targets surveillance system” to make sure the targets remained functional until their replacement.

*“We decided meetings would be every Sunday to discuss the evolution of the project and share experiences… When Tiny Targets are damaged*, *we will report it in the meeting*. *And we will take Tiny Targets from the store to replace them… Each member of the committee can also help another member to maintain his Tiny Targets…*.*” (FGD*, *KK*, *October 2017*)

Committees showed a sense of initiative and a problem-solving attitude as problems arose. For instance, in Kimwilu Kuba and in Kimwela when individuals were consistently absent from committee meetings, committees replaced them with more motivated people. For absentees with a valid excuse (e.g. health issues, travel, etc) other members helped in adjacent areas. These initiatives reduced conflicts and preserved the group dynamics and ability to achieve objectives.

Although the different committees showed some capacity of self-management, they also clearly expressed the fear of being “abandoned” and not being able to continue by themselves. They clearly mentioned the project will stop if the close support and interest from outside ceases.

*“We ask you as initiator of the project to not abandon us and to monitor our activities*. *We want you to visit us to see project progress and to avoid we get discouraged” (FGD*, *KK*, *July 2017*)

#### Committee dynamic: Cohesion, self-esteem, sense of ownership and (de) motivation

We observed cohesion between group members and a good motivation of being part of the project because they were happy to acquire new skills, new status, created new social relationships and because HAT is part of their community history’s and they wanted to eliminate it for good.

*“This project reunited us like a family*, *we did not know each other very well before and we do things together now…” (FGD*, *KK*, *October 2017*)

*“This disease caused the death of many people in recent years*. *We are happy you came to train us and gave us new tools to fight this disease…*.*We want our future generations to be free and in good health*. *We want this project to continue*.*”* (*FGD*, *KA*, *July 17*)

Despite the good dynamics and the reinforcement of their self-esteem, the volunteer aspect created frustrations that were frequently mentioned throughout the study and by all committees. In DRC, people are used to receive 5 USD when they participate in development projects in general. Even if the research team was very clear about the voluntary nature of the project, the majority of participants still expected to receive something, especially from a white person (CVK).

*“For me there is no work without reward*. *For a white*, *you have to do work first and when there are results*, *he can pay you with money*. *So you cannot say that we work without being paid*. *One day when we will be paid*, *you will see that we did not waste our time*. *(FGD*, *KK*, *October 2017*)

Another important frustration constantly reported was about the negative perception of several community members regarding volunteering.

*“In our group*, *men and women are well involved and we continue to put in effort*. *But people very often seek to discourage us*. *They mock us by saying that we lose our time with this project*, *instead of doing our field work and getting money to feed our children*, *and that we spend our time in a project for which we are not even paid*. *(FGD*, *KK*, *October 2017*)

The research team frequently received requests from committee members for financial rewards in recognition of the hard work they were doing. To respect the research protocol, this needed a lot of discussion to calm tensions and frustrations. In some rare situations, this could not be avoided: in Kimwela two members decided to keep the smartphones used to collected GPS points until receiving some payment.

### Tiny Target implementation

#### From theory to practice: A good use of acquired knowledge

*Raising awareness*: Committees insisted on the importance of transmitting their knowledge to the rest of the community before starting with the Tiny Targets deployment to ensure people did not destroy the Tiny Targets. Overall committee members stated the awareness raising activity went well, it clarified many questions and people seemed to be favourable towards the intervention.

« *Raising awareness was a success*, *because afterwards*, *everybody wanted Tiny Targets to be installed near their fields or ponds…*” (FGD, KK, July 2017)

However, initial awareness raising could not dissipate all the rumours or convince the most sceptical. After the deployment, some rumours persisted or appeared. Some people believed the Tiny Targets would scare away the game or the fish or damage the harvest. The advantage of having a permanent committee part of the community is the awareness can be continuous.

*“A woman told me the peanuts harvest was bad this year because the Tiny Targets captured the Mamiwata (ed*: *water spirit providing*, *amongst other things*, *a good harvest)*. *I told her all the villages had a bad peanut harvest*, *even the ones who did not put up Tiny Targets*, *and I explained again the importance of the Tiny Targets and she understood” (KK*, *FGD*, *February 2018*)

**Fig 5:**
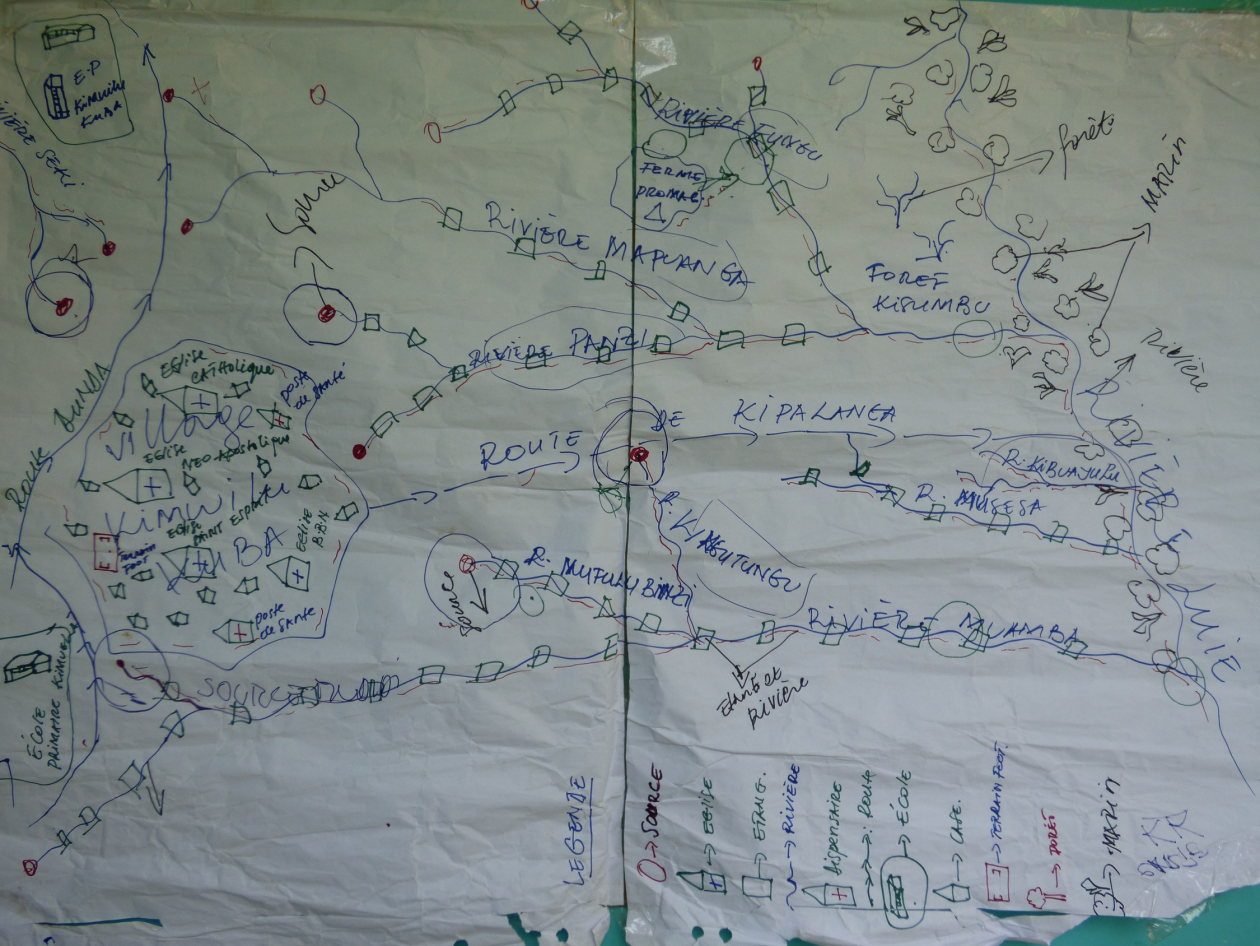
Village map drawn by Kimwilu Kuba Vector Control Committee members: Distribution of the ponds network and perceived contact points. (Credit: CVK).

*Need assessment*: Committees identified the places where they reported being bitten by tsetse flies as the place needed to be covered. This corresponded to their complex network of ponds, in the valley. To plan the Tiny Targets deployment, they first drew a map of the community area and they demonstrated a clear spatial understanding of their surrounding area and its limits (Fig 5).

However, participants over-estimated the numbers of targets needed because they over-estimated the distance of their different ponds networks. Consequently, Tiny Targets were placed too close to each other (40 Tiny Targets per linear km instead of the 20 recommended). This was corrected in the second deployment with advice from the research team.

*Tiny Targets Deployment*: Committees divided the local area into sub-zones and they divided the work of assembling and deploying the Tiny Targets amongst committee members. The main challenge encountered was the dense vegetation which restricted access and the occasional need to wade through water. They reported that the work was physically demanding, especially in the dry season when it was difficult to plant the supporting sticks into the hard ground.

**Fig 6:**
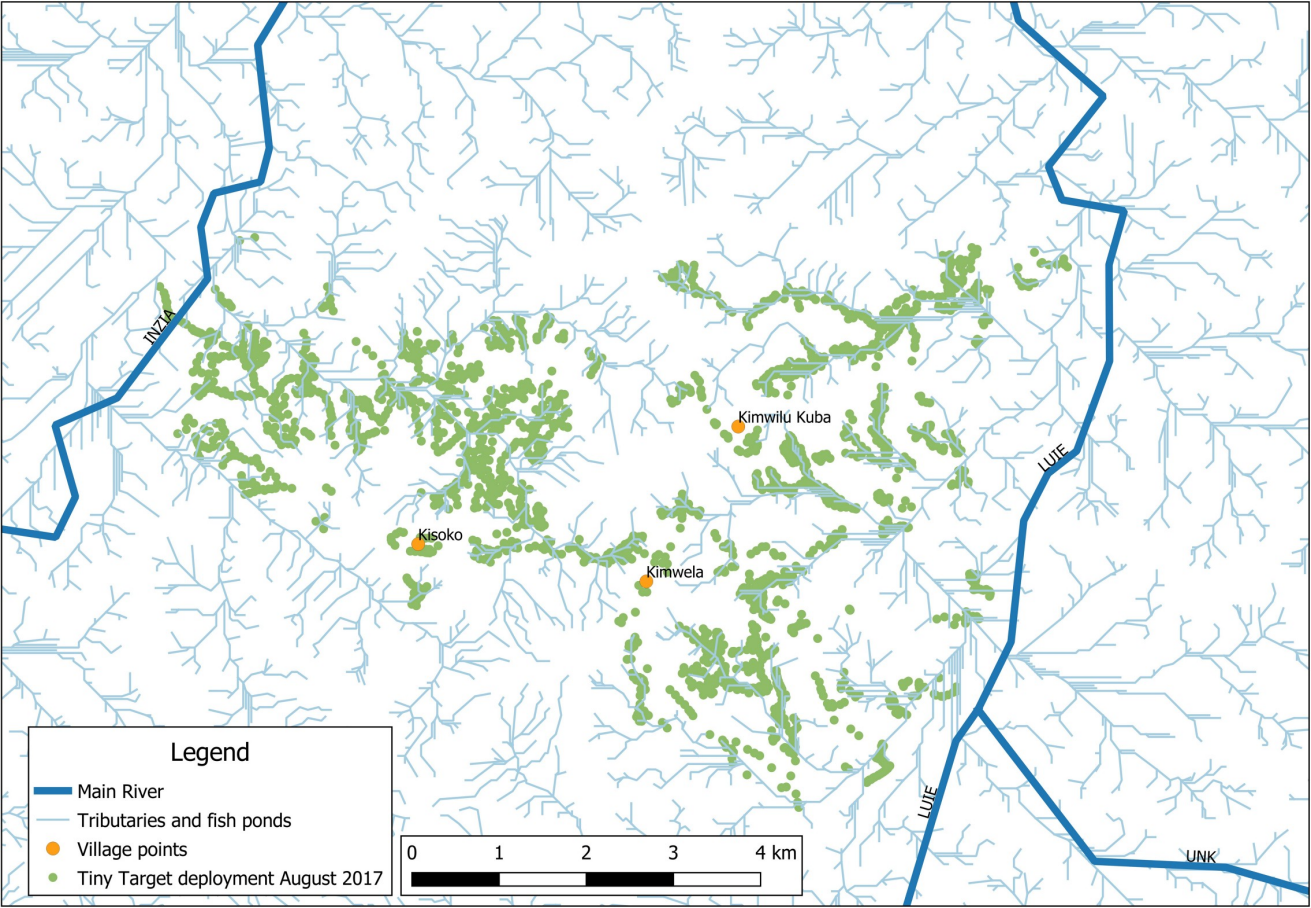
Three Pilot village committee first Tiny Targets deployment (August 2017): GPS device. (Original work: CVK & RS).

In total, 2429 Tiny Targets were deployed during the first deployment covering an area of approximately 32 km^2^ representing 75 Tiny Targets per km^2^ (Fig 6). During the second deployment a total of 848 Tiny Targets were deployed, 26 per km^2^.

An external team made a post-deployment evaluation of target quality in the field and assessed that 16 Tiny Targets (0.7%) out of the 2,244 were not functional. The cause of the malfunctions was damage caused by either weather conditions or animals. Some community members reported some Tiny Targets were missing due to theft by neighbouring villages who wanted them in their own territory.

#### Duration of a Community based Vector Control Activity: A slow process

**Fig 7:**
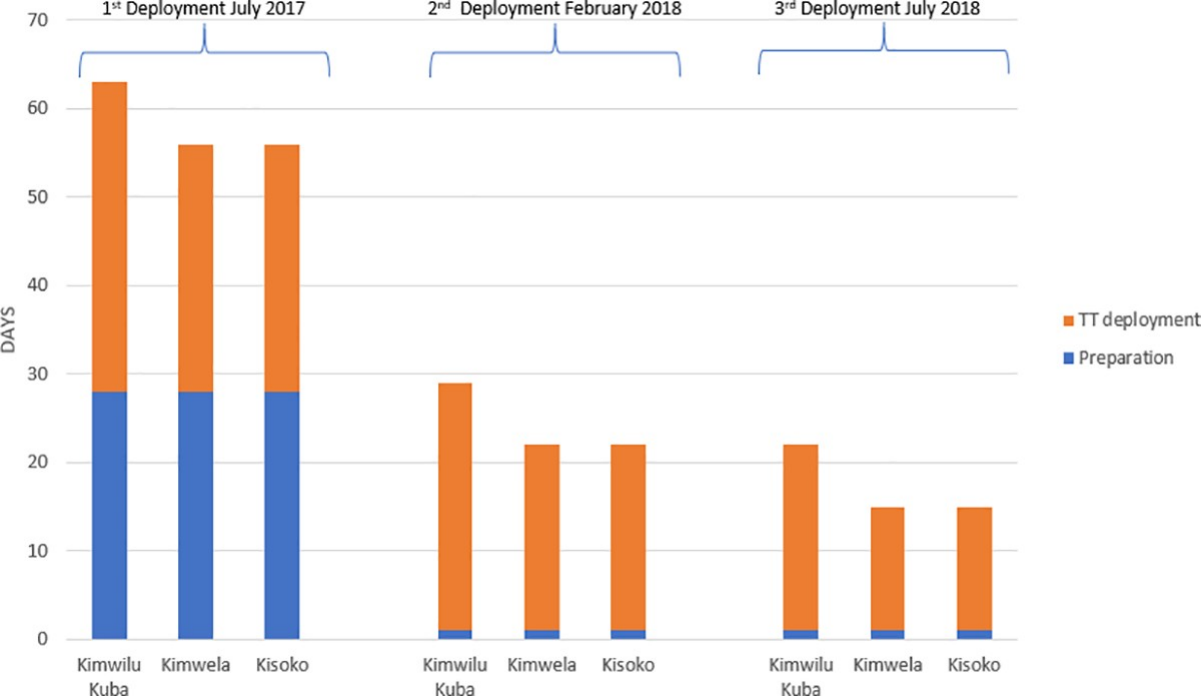
Duration of the vector control activity by Villages and by Tiny Targets deployment: Preparation and Tiny Targets deployment. (Original work: CVK).

For the first deployment, the preparation phase was particularly long as training was necessary and meetings were conducted for research purposes. Also, as the activity was on a voluntary basis, people had to do it during their free time, corresponding to about two days a week. The second and third deployment were faster to operate as there were fewer Tiny Targets to deploy and committee members had acquired skills and felt more confident (Fig 7). However, when comparing the output to a similar activity operated by the expert-led vector control team the same deployment would have been taken only 3 days [27].

### Maintenance and Evaluation system

#### Community’s evaluation indicators VS quantitative data collection

The data collection of Tiny Targets placement and quality was problematic. For the first deployment an external team recorded the location of Tiny Targets with a GPS. However, this was not well received by the committee.

“*Papa X reported the work was doubled because they had to accompany the external evaluation team to the Tiny Targets*. *He said it would have been better to take the GPS points at the same time as deploying them*. *Also*, *some remarks were made about the fact that the external team was from Mosango and not from the area*, *and this is their project*. *They wanted to do it themselves next time*.*” (extract from field notes about KO*, *October 2017*.)

For the second deployment, committees collected GPS points of their Tiny Targets with smartphones using the ODK application which is simpler to use than a GPS. However, relatively few people were able to successfully use the application and data produced were incomplete. At the third deployment, some refused to do it again because they considered it useless.

“*Kisoko refused to collect the GPS data*. *The chief told us that committee members complained it was complex and hard work for something they don’t really consider as a useful indicator*, *because they already know that targets are covering their lands well*. *GPS points and a georeferenced map seemed to be meaningless for communities” (extract of field notes about KO*, *February 2018*)

Communities have a different perception of how to assess Tiny Targets deployment effectiveness. During an evaluation meeting, committees established their own criteria to evaluate their activity such as: acquired know-how on proper deployment, target coverage, sustained actions and perceived efficacy. All villages judged the project as very effective based on their criteria:

*“We know the deployment is effective because we were taught about how to do it and we maintain the Tiny Targets” (FGD*, *KA*, *October 2017*)

*“I wanted to say that Papa X from the farm X was very happy with the Tiny Targets in the ponds because he said he does not get bitten anymore*. *It is very effective” (FGD*, *KK October 2017*)

*“To be sure it is effective*, *we need to forget no one*, *to include all the clans*, *we need to cover all areas where people get bitten*, *only this way we could eliminate sleeping sickness in our village” (FGD*, *KO*, *October 2018*)

*“At the beginning*, *I was worry because you told us we won’t catch flies with Tiny Targets so I was doubting the effectiveness*, *but I have found some dead flies near the Tiny Targets*, *so now I have no doubt” (FGD*,*KK*, *October 2017*)

#### Maintaining tiny targets standing

Committees had organised a surveillance system. Some Tiny Targets were checked daily if they were in places where committee members worked. Otherwise they were checked at monthly intervals. Children were asked to alert adults if they saw a target that needed maintenance. All communities received a stock of 100 Tiny Targets to replace damaged or missing targets. The main maintenance activity was clearance of vegetation. In six months, after the first deployment, they replaced 32 targets out of the 2429.

## Discussion

Our research provides evidence of the feasibility of community participation in tsetse control, in line with previous studies [15, 16, 19, 22, 28–33]. Studies on operational feasibility of community participation for tsetse control in DRC are very scarce in the literature, although DRC is the country most affected by HAT. This study contributes to bridging this gap. In addition, Tiny Targets are a recently developed tsetse control tool and only one study has previously assessed the feasibility of a community-based approach applying this new technology [29].

Our study showed people were motivated to participate in a project to control HAT due to their past experiences, participants were enthusiastic regarding new social relations and social status provided by the experience and felt they gained new skills and empowerment. They also showed a good application of the acquired knowledge and were proactive in taking initiatives and decisions. Exactly as described in Kovacic’s research [29], creating functional community guidance and organisation resulted in an effective deployment in tsetse habitats and with a good use of the Tiny Targets. Studies already demonstrated the efficacy of Tiny Targets [1], therefore if they are placed, used and maintained correctly, we can safely assume that the action will lead to vector reduction. In addition, our study provided evidence that acceptability of the targets by the community can improve deployment quality by preventing damage, conflicts and by encouraging non-committee members to feel involved and participate by maintaining Tiny Targets.

Although our pilot CB-approach largely worked, there were some operational problems that proved difficult to overcome. The research team successfully supported local communities to lead project implementation. However, it was difficult to assure community representation inside the committees. The village chief maintained control of the committee member selection and hence pre-existing community participation values and patterns were applied unaltered. Probably if more time were dedicated to this research it would have been possible to better understand community sub-group dynamics and to develop a more inclusive and participative strategy to create better balanced in committees.

The efficiency of the Tiny Targets deployment was also suboptimal in comparison with an expert-led approach. Tiny Targets were placed along community fishponds which correspond to tsetse habitat, but in excessive numbers, explained by the existing social logic applied by committee members. Fishponds are numerous, close to each other and divided according to clans and heritage. To avoid conflicts, committee members tried to assure that all social groups of the community were covered resulting in an excessive number being deployed.

As Kovacic observed in Uganda [29], daily community field work and tasks or specific events such as religious ceremonies or funerals affected the deployment. The Tiny Targets deployment process was slow and very time-consuming compared to the expert-led approach because a community-based approach needs to adapt to the community rhythm. In addition, frustrations linked to the volunteer aspect created another time and resource constraint. Constant discussions between the committees and the research team were necessary to maintain motivation in a context where usually community members receive financial compensation from participating in development projects. Finally the problem faced with GPS data collection was also an important operational constraint resulting from a disequilibrium between community empowerment objective and programmatic needs to measure results.

Based on the operational challenges and constraints discussed above we can legitimately ask if our CB-approach can contribute to the elimination of sleeping sickness. This study demonstrated that the CB-approach is feasible at a village-based or the so-called contact points level- i.e. where people are most likely to get bitten by tsetse. However, previous studies have demonstrated that covering contact points only is not enough to maintain adequate suppression of the tsetse population because of reinvasion pressure as tsetse move in from neighbouring uncontrolled and infested areas–as the main rivers will not receive control [1, 14, 34]. Differing to Kovacic’s conclusion, in DRC we do not believe the CB-Approach can substitute for the expert-led approach of deploying targets on the main rivers [29]. Unlike northern Uganda, villages in Kwilu are not close or accessible to the main rivers, which are difficult to reach and are not highly frequented by the community members; people prefer smaller tributaries and streams for water and washing. This difficulty of reaching the major rivers in DRC is precisely the reason why the expert-led vector control activity is done by canoe. Putting communities collectively in charge of a vector control project configured this way would be too challenging, not only logistically but also socially speaking. The community territory represents a puzzle of chiefdoms with territory rivalries.

On the other hand, the CB-approach can complement to the expert-led approach. Particularly in the fishponds; these have highly suitable habitat for tsetse and are not navigable by canoe, but they are visited frequently by community members. We argue that these pond areas can most effectively be covered with the participation of the community. Therefore a mixed approach presents the best option to reach elimination. Nonetheless, we recommend that if a CB-approach were adopted it will need some operational modifications when compared to our pilot project. In particular, we suggest that better use could be made of existing community structures, recording locations of targets using GPSs should be simplified and some incentives are probably required.

Several questions remain unanswered. It is not clear up to what scale the community-based approach is manageable. The feasibility of a scale-up by Kovacic in Uganda was based on 10 villages scale and over 50 km^2^ [29]. In the literature, the biggest scale we could find was the involvement of 54 villages in Ivory Coast covering 1,500 km^2^ surface [17]. Compared to these experiences, the zone to be covered by vector control in the ongoing programme in DRC is immense (13,000 km^2^), including more than 700 villages with very poor access. We also need to evaluate whether this approach is transferable to all the different geographical and cultural contexts present in Kwilu. Finally we need to see if communities will commit and stay motivated for a sufficient duration to achieve tsetse control effectively. The current study provided evidence that the nuisance of tsetse flies was a motivating factor for community participation. This is potentially problematic as authors have previously reported that when the nuisance of the tsetse decreased, people tended to lose interest in tsetse control [15]. In addition, it would be interesting to explore whether a community-based approach can be maintained over a longer time period with ever decreasing research presence. We have focussed on the acceptability of the CB-approach. It would also be helpful to monitor community acceptability regarding the expert-led Tiny Targets deployment compared to the community-based approach to better document benefits of community participation in vector control.

### Limitations

Our study was geographically limited and even if it was enough to draw useful conclusions, generalisation still needs to be done with caution for future large-scale operations. We are conscious that the presence of the principal investigator (CVK) will have stimulated curiosity in the village and could have enhanced adherence and involuntarily influenced discourses. We are also conscious that participants may have thought that CVK was part of the decisional structure of the National Sleeping Sickness Programme and this could have generated some expectations of incentives or other advantages. Finally, in this study we were not able to explore the full economic cost of deploying tiny targets via a community participation approach as compared to an expert led alternative. Thus, whilst we can conclude that community-led deployment approaches are feasible within the study setting, we cannot yet determine if they are cost-effective.

## Conclusion

It is feasible to implement the Tiny Target technology for tsetse control through a community-based approach. However, such community-based approach demands support, time and adaptation to the community rhythm. These represent real constraints in the short-term context of HAT elimination and the expert-led approach seems to be more adapted to the timetable and scale of the elimination agenda. However, community-based vector control also presents some undoubtable advantages such as covering contact points, better maintenance of Tiny Targets and enhanced community acceptance of the intervention. Also, in our study area, some specific human-made ecosystems, fishponds, are transmission hotspots that are difficult for the expert-led approach to access. Community-based approaches for a tsetse control strategy in DRC are feasible and provide an additional tool for the overall strategy for g-HAT elimination.
